# A rare case of multiple phosphaturic mesenchymal tumors along a tendon sheath inducing osteomalacia

**DOI:** 10.1186/s12891-017-1446-z

**Published:** 2017-02-13

**Authors:** Ryuta Arai, Tomohiro Onodera, Mohamad Alaa Terkawi, Tomoko Mitsuhashi, Eiji Kondo, Norimasa Iwasaki

**Affiliations:** 1 Departments of Orthopaedic Surgery, Hokkaido University Graduate School of Medicine, Kita-15, Nishi-7, Kita-ku, Sapporo, Hokkaido 060-8638 Japan; 2 Department of Surgical Pathology, Hokkaido University Hospital, Kita-14, Nish-5, Kita-ku, Sapporo, Hokkaido 060-8648 Japan; 3 Department of Advanced Therapeutic Research for Sports Medicine, Hokkaido University Graduate School of Medicine, Kita-15, Nish-7, Kita-ku, Sapporo, Hokkaido 060-8638 Japan

**Keywords:** Tumor-induced osteomalacia, Multiple phosphaturic mesenchymal tumor, Fibroblast growth factor 23, Hypophosphatemia, Systemic venous sampling, Case report

## Abstract

**Background:**

Tumor-induced osteomalacia (TIO) is a rare paraneoplastic syndrome characterized by renal phosphate wasting, hypophosphatemia, reduction of 1,25-dihydroxyl vitamin D, and bone calcification disorders. Tumors associated with TIO are typically phosphaturic mesenchymal tumors that are bone and soft tissue origin and often present as a solitary tumor. The high production of fibroblast growth factor 23 (FGF23) by the tumor is believed to be the causative factor responsible for the impaired renal tubular phosphate reabsorption, hypophosphatemia and osteomalacia. Complete removal of the tumors by surgery is the most effective procedure for treatment. Identification of the tumors by advanced imaging techniques is difficult because TIO is small and exist within bone and soft tissue. However, systemic venous sampling has been frequently reported to be useful for diagnosing TIO patients.

**Case presentation:**

We experienced a case of 39-year-old male with diffuse bone pain and multiple fragility fractures caused by multiple FGF23-secreting tumors found in the hallux. Laboratory testing showed hypophosphatemia due to renal phosphate wasting and high levels of serum FGF23. Contrast-enhanced MRI showed three soft tissue tumors and an intraosseous tumor located in the right hallux. Systemic venous sampling of FGF23 revealed an elevation in the right common iliac vein and external iliac vein, which suggested that the tumors in the right hallux were responsible for overproduction of FGF23. Thereafter, these tumors were surgically removed and subjected to histopathological examinations. The three soft tissue tumors were diagnosed as phosphaturic mesenchymal tumors, which are known to be responsible for TIO. The fourth tumor had no tumor structure and was consisting of hyaline cartilage and bone tissue. Immediately after surgery, we noted a sharply decrease in serum level of FGF23, associated with an improved hypophosphatemia and a gradual relief of systematic pain that disappeared within two months of surgery.

**Conclusion:**

The authors reported an unusual case of osteomalacia induced by multiple phosphaturic mesenchymal tumors located in the hallux. Definition of tumors localization by systemic venous sampling led to successful treatment and cure this patient. The presence of osteochondral tissues in the intraosseous tumor might be developed from undifferentiated mesenchymal cells due to high level of FGF23 produced by phosphaturic mesenchymal tumors.

## Background

Tumor-induced osteomalacia (TIO) is a rare paraneoplastic syndrome characterized by overproduction of fibroblast growth factor 23 (FGF23), defect in vitamin D metabolism, chronic hypophosphatemia, and disorder in bone calcification. Phosphaturic mesenchymal tumors associated with osteomalacia produce high FGF23, which inhibits phosphate transport in renal proximal tubule epithelial cells, resulting in renal phosphate wasting [1–3]. Moreover, FGF23 reduces circulating 1,25-dihydroxyl vitamin D through suppressing the production of 1-alpha hydroxylase. The deficiency of the circulating levels of phosphorus and 1,25-dihydroxyl vitamin D impairs mineralization of osteoid matrix in mature bone leading to a defect in the bone-building process.

Muscle weakness, myalgia and bone pain are the major clinical symptoms of TIO, and patients are commonly misdiagnosed with other illnesses such as rheumatologic diseases or psychiatric disorders [4, 5]. Patients with advanced osteomalacia may suffer from fragility fracture and gait disturbance due to the impairment in bone quality. Multiple fragility fractures are often occurred in ribs, vertebral bodies, and femoral neck [6, 7]. Complete surgical resection of the tumors cures TIO dramatically improves the metabolism of phosphorus and restores the normal levels of circuiting vitamin D, resulting in rapid resolution of clinical symptoms within a few weeks. However, diagnosis of TIO is always challenging and is delayed in most cases due to the small size and slow growth of these tumors. High-resolution magnetic resonance imaging (MRI) and F-18 fluorodeoxyglucose positron emission tomography (FDGF-PET) are proposed to be beneficial modalities to define the location of the tumor. Moreover, DOTATOC-PET/CT has been reported to be a feasible option for localizing causative tumors in patients with TIO [8], but this modality is not approved in our county. Systemic venous sampling that detects the excess production of FGF23 in the culprit tumor has recently emerged as greater technique for precise definition of tumor location [9, 10].

Phosphaturic mesenchymal tumors associated with TIO are polymorphous neoplasms that are originated from bone or soft tissue. These tumors are typically not malignant composing of spindled-stellate cells that appears normochromatic, small with indistinct nucleoli. In the most of published cases, nuclear atypia and mitotic activity are low [11–13]. Generally, they are nonaggressive tumors and appear solitary throughout the affected tissue. A rare case of two phosphaturic mesenchymal tumors has been earlier reported by Nathan et al. In that published case, single tumor was initially detected in the tibia, and second tumor was raised two years later in the maxillary sinus [14]. In a related study, Higley et al. [15] have showed two unusual cases of TIO characterized by locally aggressive and multifocal phosphaturic mesenchymal tumors. Herein, the authors reported a case of three phosphaturic mesenchymal tumors along the tendon sheath in the hallux with no malignancy. Resecting these tumors resulted in an elevation in serum level of phosphorus accompanied with complete resolution of clinical symptoms.

## Case presentation

**Fig. 1:**
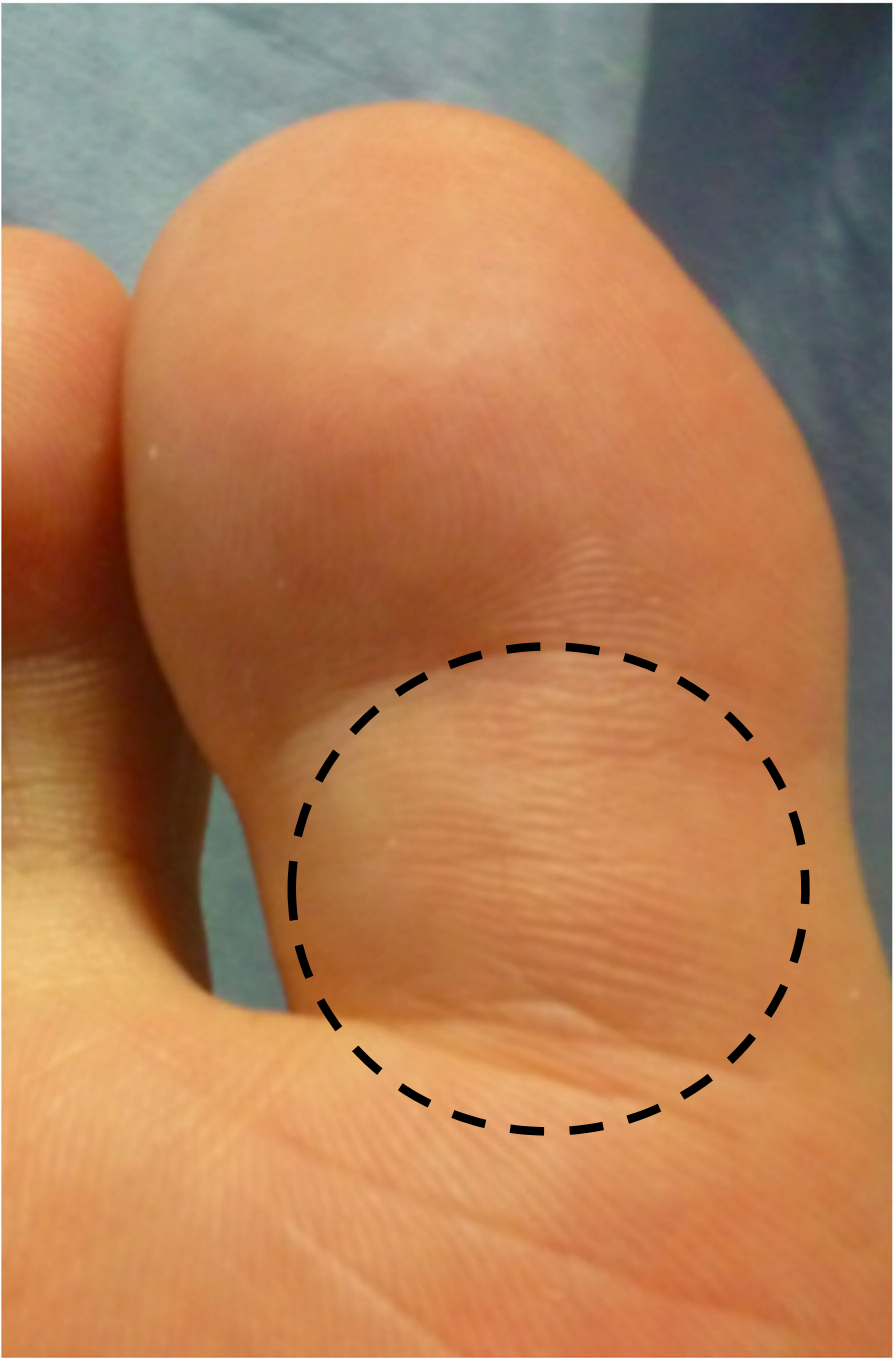
Soft tissue tumor in plantar side of the right hallux

**Fig. 2:**
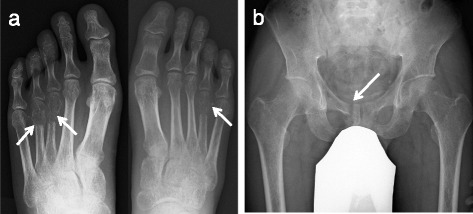
Radiographic observations. **a** AP radiograph shows cystic radiolucent shadow in right fourth metatarsal bone (*arrows*) and left third and fourth metatarsal bone (*arrows*). **b** AP radiograph shows cystic radiolucent shadow in right pubis (*arrow*)

**Fig. 3:**
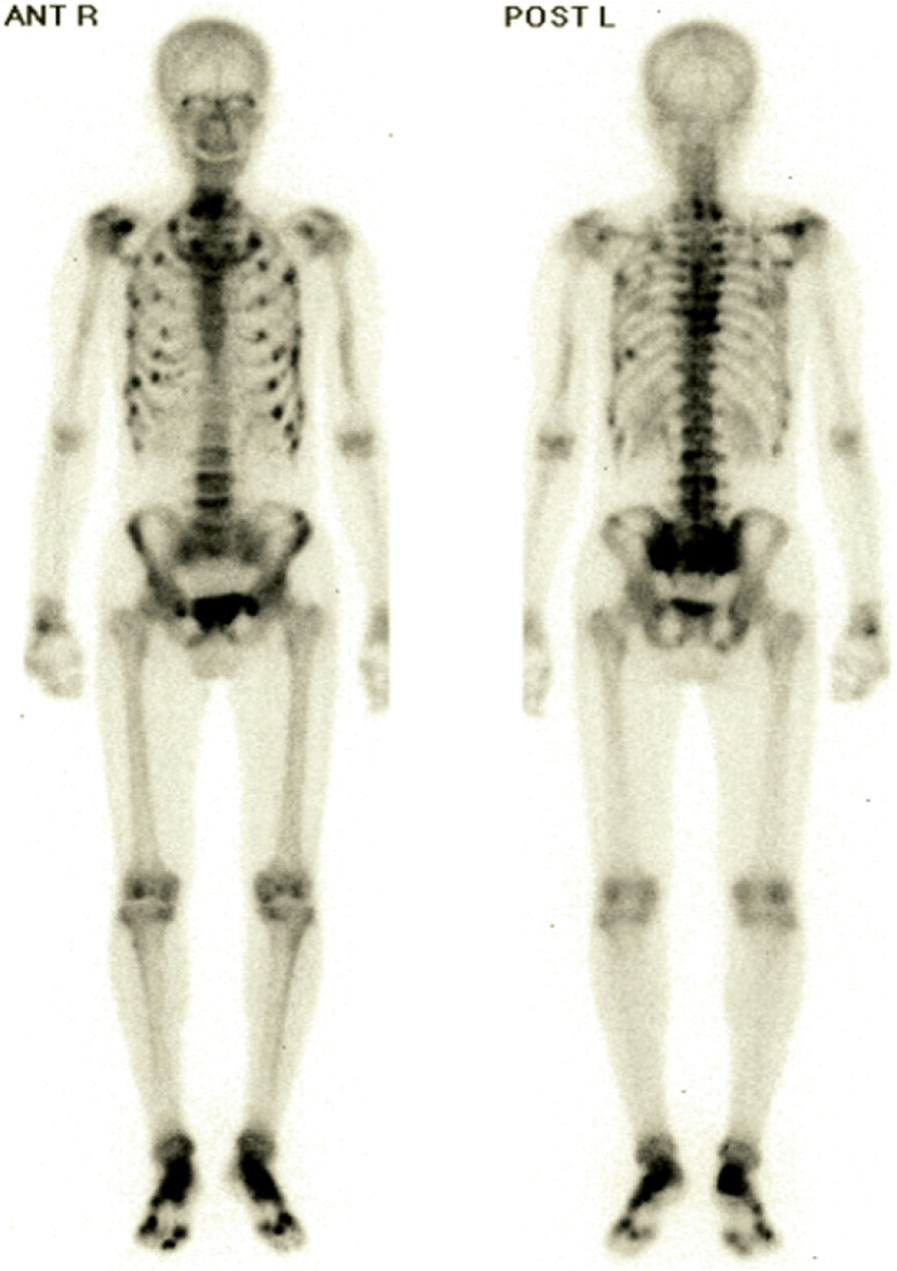
Bone scan observation. Image shows an increased uptake in the bilateral right pubis, bilateral tarsus, right fourth metatarsal bone, left third and fourth metatarsal bone

**Fig. 4:**
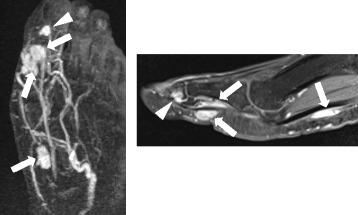
Magnetic resonance imaging. Gadolinium-enhanced T2 weighted magnetic resonance imaging shows three soft tissue tumor presented along right flexor hallucis longus muscle (*arrow*) and one tumor in right first distal phalanx (*arrowhead*)

**Fig. 5:**
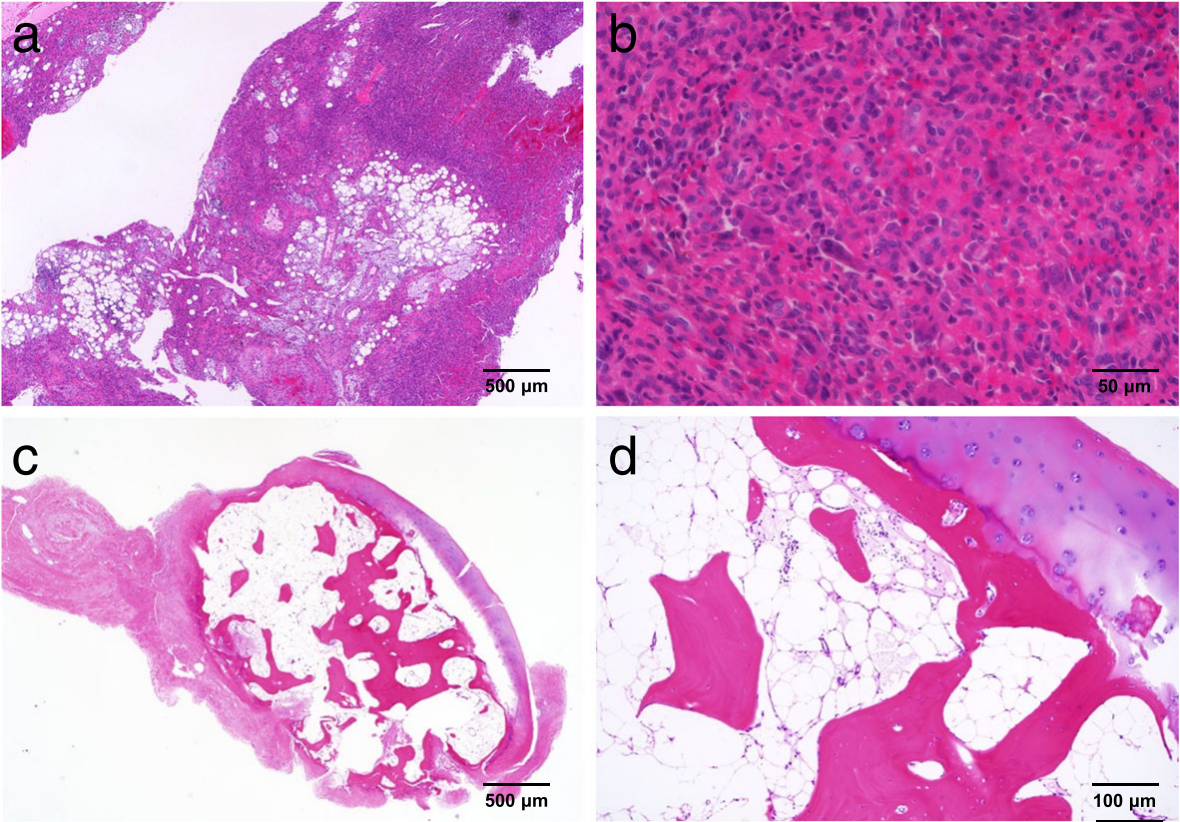
Histopathological findings of the tumors. **a** Low power view of HE-stained sections of the mesenchymal tumors found in flexor halluces longus muscle tendon. **b** Higher magnification image for the above section that shows oval-shaped mesenchymal cells densely populated in fibrous background, osteoclast-like giant cells and histiocytes associated with haemorrhage and hemosiderin deposition. Mitotic activity and necrosis are absent. **c** Low power view of HE-stained sections of the tissue excised from distal phalanx. **d** Higher magnification image of the above tissue that shows hyaline cartilage and bone tissue

A 39-years old male had a low back pain without any particular cause, following chest pain, right hip pain, and bilateral foot pain. One year after these symptoms, he experienced enlarging elastic soft masses in plantar side of the right hallux (Fig. 1). He had no remarkable family history of metabolic bone diseases. He was 172.6 cm tall and 63.8 kg weight (Body mass index = 21.4 kg/m^2^) at the time of his first visit. Laboratory testing of serum samples revealed normal calcium level of 9.3 mg/dl (normal range: 8.7-10.3 mg/dl), low phosphorus level of 1.9 mg/dl (normal range: 2.5-4.5 mg/dl). Alkaline phosphatase was elevated as noted to be 1245 U/l (normal range: 115–359 U/l). Serum level of FGF23 (215 pg/ml) was at least 4-fold higher normal range (normal range: 10–50 pg/ml). Urine testing revealed an elevation in phosphorus concentration coincident a reduction in tubular phosphate reabsorption. Plain radiographic images showed cystic radiolucent shadow in right fourth metatarsal bone, left third and fourth metatarsal bone, and in right pubis (Fig. 2). Bone scanning revealed an increased uptake in the bilateral rib, right pubis, bilateral tarsus, right fourth metatarsal bone, left third and fourth metatarsal bone (Fig. 3). Enhanced MRI showed that three soft tissue tumors were presented along with right flexor hallucis longus muscle tendon and one tumor in right first distal phalanx (Fig. 4). Taken together, this case was diagnosed as TIO and these tumors were expected to be responsible for overproduction of FGF23. To ascertain our diagnosis, systemic venous sampling for the measurement of FGF23 was conducted. Of note, serum concentrations of FGF23 in right common iliac vein and in right external iliac vein were higher than other veins (Table 1). These data indicated that tumors in right hallux detected with MRI were responsible for the osteomalacia. Thereafter, tumors of flexor tendon were resected with the wide margins. Tumor of distal phalanx found in adhesion section of flexor tendon was easily excised from surrounding bone. Histological examination of the three tumors located on flexor tendon showed oval-shaped mesenchymal cells densely populated in fibrous background. Moreover, osteoclast-like giant cells and histiocytes were sparsely found in these tumors associated with multifocal haemorrhage and hemosiderin deposition. Mitotic activity and necrosis were absent (Fig. 5a, b). On the other hand, the tissue excised from distal phalanx consisted of hyaline cartilage and bone tissue showed no tumor (Fig. 5c, d).

**Table 1:** Systemic venous sampling with measurement of FGF23

Vein	Serum FGF23 (pg/ml)
Right internal jugular	164
Left internal jugular	170
Superior vena cava	162
Right subclavian	164
Left subclavian	140
Right brachiocephalic	162
Left brachiocephalic	169
Right atrium	144
Proximal inferior vena cava	197
Right hepatic	139
Right renal	161
Left renal	131
*Right common iliac*	**221**
*Right external iliac*	**271**
Right internal iliac	121
Left common iliac	181
Left external iliac	194
Left internal iliac	180

The serum level of FGF23 dramatically decreased to be 47 pg/ml and phosphorus level returned to normal (3.9 mg/dl), within one hour after the surgery. Serum concentration of FGF23 became 10 pg/ml one day post-operation. Moreover, systematic pain was gradually improved, and disappeared two months after surgery.

## Discussion

The first case of TIO was reported in 1947 [16], and the link between phosphaturic mesenchymal tumor and osteomalacia was firstly recognized by Prader and colleagues in 1959. Furthermore, the first identification of FGF23 as the causative factor of TIO was documented in 2001 [17]. FGF23 is a member of the FGF ligand superfamily, produced by osteogenic cells, osteoblasts and osteocytes, and functions as regulator of phosphate homeostasis and transport in the kidney. The overproduction of FGF23 by phosphaturic mesenchymal tumor results in an elevation of renal phosphorus wasting and impairment of intestinal phosphorus absorption, leading to hypophosphatemia and osteomalacia. FGF23 has negative regulatory effects on circulating 1,25-dihydroxyl vitamin D by altering cellular production of 1-alpha hydroxylase and 24-hydroxylase in kidney [7]. These metabolic disorders impair mineralization of osteoid matrix in mature bone and cause a defect in the bone-building process. Therefore, patients with TIO frequently suffer from multiple fractures and generalized debilitated status.

In this report, we presented a rare case of osteomalacia induced by multiple phosphaturic mesenchymal tumors, diagnosed by MRI and systemic venous sampling, and confirmed by histopathological examination. The patient experienced a diffuse bone pain and multiple fragility fractures due to the disorder in bone metabolism and the poor mineralization of osteoid in mature bone. Laboratory abnormalities that are often associated with osteomalacia were observed in this case, including a reduction in serum phosphorus and an elevation in serum alkaline phosphatase and FGF23. Three soft tissue tumors and an intraosseous tumor were found to be located along with the right hallux. The elevated level of FGF23 in right common iliac and external iliac veins detected by systemic venous sampling suggested that tumors localized in the right hallux responsible for the osteomalacia.

Osteomalacia induced by multifocal phosphaturic mesenchymal tumors is extremely rare and most of reported TIO cases are associated with single and small, slow-growing soft tissue or bone neoplasms tumor [15]. Such attribute may delay the tumor recognition, localization and treatment. Imaging modalities including radiographs, computed tomography scans, MRI, technetium bone scanning, and positron emission tomography are routinely used for defining tumor localization. However, systemic venous sampling that detects the overproduction of FGF23 secreted by phosphaturic mesenchymal tumor has been recently recommended for the definition of tumor localization [9, 10]. In the current report, localization of phosphaturic mesenchymal tumors was defined by systemic venous sampling and MRI, and these tumors were resected with a wide surgical margin. Complete recovery with no further complexations was achieved after surgery.

Resected tumors located on right flexor hallux longus muscle tendon exhibited typical histopathological features phosphaturic mesenchymal tumors, including the numerous number of mesenchymal cells characterized by spindled to stellate shape and with small nuclei or indistinct nucleoli. Osteoclast-like giant cells, histiocyte, haemorrhage, and hemosiderosis were also present in the lesions. Additional tumor found in the right distal phalanx had no structure of mesenchymal tumor consisting of hyaline cartilage and bone tissue. The presence of osteochondral tissue in the intraosseous tumor is most likely due to the overproduction of FGF23 that triggers the differentiation of mesenchymal cells into chondrocyte and osteocyte, and promotes the proliferation of chondrocytes [18, 19].

## Conclusions

We reported a rare case of TIO caused by multiple phosphaturic mesenchymal tumors in the hallux. Surgical resection of tumors resulted in a rapid resolution of the metabolic disturbance and clinical symptoms. The osteochondral tissues found in these tumors might be originated from undifferentiated mesenchymal cells that may differentiate into chondrocyte and osteocyte by the overproduction of FGF23.

## Abbreviations

FDG-PET: F-18 fluorodeoxyglucose positron emission tomography
FGFR: Fibroblast growth factor receptor
FGF23: Fibroblast growth factor 23
MRI: Magnetic resonance imaging
TIO: Tumor-induced osteomalacia
